# Reenvisioning Traditional to Regenerative Therapeutic Advances in Managing Nonalcoholic Fatty Liver Disease in Diabetes Mellitus

**DOI:** 10.1155/2021/7692447

**Published:** 2021-11-11

**Authors:** Lung-Wen Tsai, Yi-Hsiang Lu, Rajni Dubey, Jeng-Fong Chiou

**Affiliations:** ^1^Department of Medicine Research, Taipei Medical University Hospital, Taipei 11031, Taiwan; ^2^Department of Information Technology Office, Taipei Medical University Hospital, Taipei 11031, Taiwan; ^3^Graduate Institute of Data Science, College of Management, Taipei Medical University, Taipei 11031, Taiwan; ^4^Department of Otolaryngology, Taipei Medical University Hospital, Taipei 11031, Taiwan; ^5^Department of Radiation Oncology, Taipei Medical University Hospital, Taipei 11031, Taiwan; ^6^Department of Radiology, School of Medicine, College of Medicine, Taipei Medical University, Taipei 11031, Taiwan

## Abstract

Reports indicate the increasing prevalence of liver disorders in diabetes mellitus (DM) patients. Clinically, it has also been revealed that the existence of nonalcoholic fatty liver disease (NAFLD) enhances the incidence of type 2 diabetes mellitus (T2DM), while T2DM exacerbates NAFLD to extremely severe forms of steatohepatitis, cirrhosis, and hepatocellular carcinoma. This implies the coexistence and bidirectional nature of NAFLD and T2DM, which function synergistically to drive adverse consequences in clinical practice. For treatment of such comorbid state, though the existing practices such as lifestyle management, traditional Chinese medicines (TCM), and pharmaceuticals have offered somewhat relief, the debate continues about the optimal therapeutic impacts. Recent developments in the field of tissue engineering have led to a renewed interest in novel biomaterial alternatives such as stem cells. This might be attributable to their differentiation potential towards hepatic and pancreatic lineage. These cellular therapies could be further complemented by platelet-derived biomaterials, TCM formulations, or any specific drug. Based on these abovementioned approaches, we aimed to comprehensively analyze various preclinical and clinical studies from traditional to regenerative therapeutic approaches in managing concomitant NAFLD and T2DM.

## 1. Introduction

Nonalcoholic fatty liver disease (NAFLD) is highly common in diabetes mellitus (DM), a syndrome characterized by altered glucose metabolism [1], and evidence implies that these concomitant pathologies are bidirectional [2]. Specifically, NAFLD participates in the development of type 2 DM (T2DM) by elevating glucose production in the liver and aggravating hepatic insulin resistance [3]. On the other hand, T2DM and insulin resistance stimulate an increase of free fatty acid flux from peripheral tissues to the liver, resulting in the development and progression of NAFLD. A recent United States-based study demonstrated the prevalence of advanced liver fibrosis in patients with T2DM [4]. A twofold higher prevalence of NAFLD (55.5%) in diabetes patients compared to the general population has also been reported [5]. This coincides with epidemiological characteristics of NAFLD from 1999 to 2018 in China showing a notable increasing trend of obesity [6]. A recent meta-analysis of NAFLD in China reported a 51.83% prevalence of NAFLD in patients with diabetes compared to 30.76% of healthy ones [7]. Notably, this prevalence was also found higher among obese (66.21%) than nonobese population (11.72%). Based on the abovementioned evidence, the bidirectional interaction between NAFLD and DM could be inferred.

The presence of chronic liver disease in diabetes patients significantly increases the risk of glucose intolerance and insulin resistance, which renders them vulnerable to liver fibrosis, cirrhosis, and hepatocellular carcinoma [8, 9]. Thus, it seems critical and challenging to reduce morbidity and mortality in patients with liver disease and DM, which may further complicate due to drug metabolism in the liver and risk of hepatotoxicity [10]. Notwithstanding, the treatment of DM linking liver disorders through glucose-lowering agents such as metformin, pioglitazone, GLP-1 receptor agonists, and SGLT-2 seems advantageous [9]. For most of the patients, metformin if tolerated has been recommended as an appropriate first-line therapy, excluding those having advanced liver disorder, who might be susceptible to enhanced risk of lactic acidosis [11]. According to Khan et al., metformin for chronic liver disease patient likely to be safer, with reduced dose of 1500 mg daily, and may be withdrawn in the case of declining liver or renal function [12] Specifically, long-term pioglitazone therapy has been reported safe and efficacious for the patients with T2DM and NAFLD [13]. In a similar trend, DPP-4 inhibitors like sitagliptin have been suggested effective and safe for DM patients complicated by liver injuries [14], whereas the second-line therapies GLP-1 receptor agonists and SGLT-2 inhibitors exhibit positive impact on body weight with reduced risk of hypoglycemia.

Based on limitations such as the risk of lactic acidosis and hypoglycemia associated with these oral hypoglycemic agents has prompted the scientific community to explore other safer and efficacious alternatives [15]. Regular exercise and a controlled diet have also been evidenced as somewhat effective [10]. For decades, the traditional herbs and Chinese medicines (TCM) have been shown to exert therapeutic effects in various disorders with either minimal or no side effects. These may suppress the risk of NAFLD as well as DM [16, 17]. However, to further explore the enhanced therapeutic efficacies, stem cells or platelet-based regenerative alternatives are being examined in various preclinical and clinical studies [18–20]. The stem cells through their differentiation potential towards pancreatic *β*-cells and hepatocyte lineage may also regulate glucose/lipid metabolism and exert anti-inflammatory actions [21]. Hence, the burden of liver transplantation and related risks could be considerably reduced. Nevertheless, developing regenerative therapy for NAFLD is still at the infant stage. Considering challenges and available therapeutic tools for managing concomitant NAFLD and DM, this article has extensively reviewed preclinical and clinical studies from traditional to advanced regenerative therapeutic interventions.

## 2. Pathophysiology of Hepatic Disorders and DM

**Figure 1:**
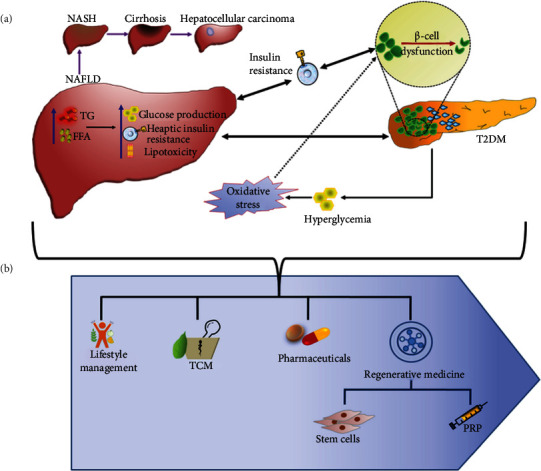
Association between the pathophysiological duo of NAFLD and DM and its treatment. (a) The mechanistic insight underlying NAFLD and DM. NAFLD participates in the development of T2DM by enhancing glucose production and insulin resistance in the liver. T2DM and systemic insulin resistance induces the initiation and progression of NAFLD by increasing levels of FFA and TG from peripheral tissues to the liver. If remain untreated, NAFLD further progresses from NASH, cirrhosis, to hepatocellular carcinoma. (b) Journey of therapeutic alternatives from traditional to regenerative medicines, including lifestyle management, TCM, pharmaceuticals, stem cells, and PRP. NAFLD: nonalcoholic fatty liver disease; FFA: free fatty acid; TG: triglycerides; NASH: nonalcoholic steatohepatitis; T2DM: type 2 diabetes mellitus; TCM: traditional Chinese medicines; PRP: platelet-rich plasma.

NAFLD is correlated to DM due to shared pathophysiological characteristics like adipose accumulation and insulin resistance (Figure 1(a)) [22]. These characteristics participate in NAFLD progression, by insulin resistance-induced excess synthesis of triglyceride, accumulation, and impaired oxidation of free fatty acid (FFA), and secretion of very-low-density lipoproteins (VLDL) resulting in severe hepatic stress. NAFLD not only contributes to the development of liver cirrhosis and cardiovascular complications but also acts as an etiological factor for cancer initiation and progression. A systemic review and meta-analysis concluded that NAFLD may trigger hepatocellular, colorectal, breast, pancreatic, prostate, and esophageal cancer [23, 24]. Though the exact mechanism underlying NAFLD-induced cancer is not well-established, the possible contributing factors may include unregulated efflux of adipokines, increased levels of IGF-1, insulin, and cytokines (TNF-*α*, IL-6), accelerated hepatocyte proliferation, lipid peroxidation, oxidative stress, DNA damage, and lipotoxicity [25–29]. The cytokines TNF-*α* and IL-6 mediate its antiapoptotic impact through activating STAT3 oncogenic transcription factors ensuing carcinoma [30]. Furthermore, the irregular lipid metabolism in NAFLD inhibits the influx of CD4 + T cells resulting in the accumulation of CD8+ T cells in the liver and the development of hepatic cancer [31]. It has been further established that NAFLD could promote the expression of IL-1*β*, VEGF, and NOD-like receptor C4 in tumor-associated macrophages and accelerate the growth of the liver tumor [32].

Similarly, hepatogenous diabetes (HD) has been evidenced by the progression of irregular insulin clearance and *β*-pancreatic cell apoptosis [33]. The presence of liver disorders disrupts glucose metabolism due to insulin resistance and impaired sensitivity of pancreatic islet *β*-cells [34, 35]. Initially, insulin resistance and glucose intolerance occur at the initial stage of HD; however, with its progression, the manifestation of diabetic symptoms becomes clinically distinct. HD is also associated with a low incidence of microangiopathy, reduced response to antiviral treatment, and complicated treatment procedure due to cirrhosis and liver toxicity of drugs. It is also a causative factor for the progression of hepatocellular carcinoma [35, 36], which might be ascribed to polymorphisms in TCF7L2 rs290487 and rs6585194 gene along with the presence of SNPs rs290481, rs290487, and rs29048 at 3′ end of TCF7L2 gene [37]. In addition, HD induces secretagogue of adipokines such as adiponectin, leptin, HGF, TNF-*α*, TGF-*β*1, and resistin, resulting in liver fibrosis and inflammation [38–40]. The mortality in HD patients also increases due to immunosuppressive activity and increased risk of infection [41].

Notably, viral infections such as Coxsackievirus B, rotavirus, mump virus, the rubella virus, and cytomegalovirus may cause T1DM [42], whereas hepatitis C viral infection enhances the risk of T2DM with the escalated frequency of fibrosis, cirrhosis, and hepatocellular carcinoma [43]. Though the association of DM with the severe progression of hepatic injury and carcinoma along with other complications poses the therapeutic challenge, the research advances from traditional to regenerative treatment regimens (Figure 1(b)) indicate considerable successes, which have been extensively reviewed in our next sections.

## 3. Treatment Strategies for Concomitant Liver Disease and Diabetes

### 3.1. Lifestyle Management

As NAFLD and DM are associated with food habits, type and pattern of fat consumption, exercise and daily life activity, and careful management of these lifestyle-related factors are imperative [44–46]. Therefore, the recommendation of lifestyle management is considered as a first-line therapeutic approach for NAFLD, DM, liver infection, and other severe liver disorders [47]. Regarding NAFLD, the recommended guidelines for dietary changes include reduction of saturated fat intake to <7% of total calories, trans-fat and dietary cholesterol < 200 mg per day, and total fat at 25%-35% of total calories [48]. According to the American Association for the Study of Liver Diseases (AASLD), reducing body weight by at least 3%-5% may improve hepatic steatosis, while body weight reduction to ≥7% could improve histological characteristics of NASH including fibrosis [49]. Similarly, the Korean Association for the Study of the Liver (KASL) has also recommended a body weight reduction of 7%-10% for improving NAFLD [50]. A long-term clinical follow-up of lifestyle-related intervention for 6 years showed a significantly reduced risk of DM [51]. This might further suppress insulin resistance and hence the occurrence of NAFLD and its progression to other severe liver diseases. It is also known that a BMI higher than 21 is associated with an increased risk of DM [52]. Therefore, the initial weight loss and exercise seem crucial in controlling DM [53, 54], and clinical studies have demonstrated that combined lifestyle intervention and metformin-mediated weight loss significantly reduced the incidence of DM [55, 56]. In addition to regular exercise, a very low energy diet is also effective in weight loss, glycemic control, and regulation of lipid metabolism among overweight T2DM patients [57]. In addition, a Mediterranean diet rich in fruit and vegetable may prevent DM by antioxidative stress, anti-inflammatory, and anti-insulin resistance activities [44, 58]. Thus, well-planned lifestyle changes may be an effective preventive tool for DM and its complications.

### 3.2. Pharmaceutical Interventions

The pharmaceutical intervention is the most common approach to control the progression of NALFD and DM [59]. A reduction of 1% glycated hemoglobin has the potential to diminish 35% microvascular complications and 25% diabetes-related mortality [60]. Thiazolidinediones or glitazones are the agonists of peroxisome proliferator-activated receptors (PPAR), which play an important role in glucose and lipid homeostasis, in addition to suppressing inflammation and fibrogenesis [61]. Hence, these agents could inhibit the accumulation of hepatic triglyceride, a hallmark of the development of NAFLD. In various clinical trials, the approved glitazones have not been only reported to benefit diabetes but also improve histological lesions of NASH [13, 62, 63]. Metformin is a primary drug to lower blood glucose and glycated hemoglobin (HbA1c) in T2DM [64]. It could also modify gut microbiota and actuate mucosal AMP-activated protein kinase in hepatocytes, the combined effect of which could lower the levels of lipopolysaccharides [65, 66]. Besides, insulin is widely used biologics to address the therapeutic requirement among T1DM patients, and recent progress towards the development of oral insulin has opened the way to overcome repeated pain exposure by injectable insulin [67]. Notwithstanding, the repeated long-term exposure to external insulin may slowly develop insulin resistance. Sulfonylureas and meglitinides are second-line drugs, which stimulate *β*-cells to secrete insulin mediated by pancreatic ATP-sensitive potassium channels [68, 69]. However, long-term exposure to sulfonylureas and meglitinides might cause weight gain, hypoglycemia, and deterioration in their efficacy [64]. In the line, thiazolidinediones, sodium-glucose co-transporter-2 inhibitors, and dipeptidyl peptidase-4 inhibitors are other considerable groups of drugs for DM treatment. Glucagon-like peptide-1 (GLP-1), a peptide-based alternative, has also been found effective due to its insulin enhancing, glucagon lowering, and appetite-reducing potential [70]. The GLP-1 also lowers the risk of endothelial dysfunction, myocardial ischemia, and renal failure [64, 71]. Though there seems to be a wide spectrum availability of therapeutic agents for DM treatment, in the light of adverse reactions associated with their long-term use, the urgent search for other suitable alternatives is a pressing need.

### 3.3. The Spectrum of Traditional Chinese Medicine (TCM)

For many generations, TCMs have been implicated in the treatment of various disorders due to their considerable efficacy with minimum or no adverse effect. With the advent of time, TCM-based DM treatment has been changed following social environment and lifestyle [72]. Clinical evidence reveals that Chinese herbs tian qi and tang-min-ling may significantly reduce the fasting blood glucose and glycosylated hemoglobin levels and improve insulin resistance and function of pancreatic *β*-cell [73, 74]. Therefore, in this article, we have comprehensively reviewed TCM decoctions/concoctions and independent herbs in offering therapeutic relief from concomitant DM and NAFLD.

### 3.4. Rehmannia Six Formula (RF)

RF, a concoction of six herbs, i.e., *Rehmannia glutinosa*, Fructus Corni, *Dioscorea* sp. (*D. alata*, *D. opposita*, *D. batatas*), *Poria cocos*, *Alisma sp.* (*A. orientalis*, *A. plantago aquatica*), and *Paeonia suffruticosa*, may effectively regulate blood glucose level through its strong antioxidant and anti-inflammatory actions [75]. This implies that RF could be beneficial in addressing DM and its complications by suppressing insulin resistance. The earlier onset and prolonged duration of T2DM may portend the possibility of developing NAFLD [76]; thus, the TCMs controlling NAFLD may be combined with novel herbal formulation to establish therapy for concomitant NAFLD and DM. Similarly, the other form of RF, i.e., Liuwei Dihuang decoction, may improve steatosis-associated histologic changes in the liver by inhibiting insulin resistance by regulating PI3K/Akt signaling pathway [77].

### 3.5. Shenling Baizhu San

Shenling Baizhu San is a promising TCM alternative for suppressing NAFLD via targeting glycerophospholipid and glycerolipid along with inhibition of SIRT1 in rat liver [78]. It could also regulate the expression of miRNAs such as miR-155-5p, miR-146b-5p, miR-132-3p, and miR-34a-5p to undermine the progression of NAFLD [79]. In a combinatorial approach, Shenling Baizhu San when mixed with Chaihu-Shugan-San effectually lowered the serum concentration of TNF-*α* and IL-6 [80]. This is indicative of suppressed inflammatory profile and improved lipid metabolism through regulating the expression of molecules involved in the p38 MAPK signal pathway in the rat model of NAFLD progression.

### 3.6. Lingguizhugan, Xiaochaihu, and ShengMai-Yin and Ganmaidazao: Decoctions

In a study, Lingguizhugan decoction of *Poria*, *Ramulus Cinnamomi*, *Rhizoma Atractylodis Macrocephalae*, and *Radix Glycyrrhizae* ameliorated phenotypic properties of NAFLD rats by regulating pathways of insulin resistance and lipid metabolisms such as PI3K-Akt and AMPK [81]. Lingguizhugan could also mitigate NAFLD by suppressing the expression of *INSIG1* and *LPIN1* genes, indicating decreased oxidative stress, cholesterol biosynthesis, and triglyceride accumulation in the liver [82]. Among various decoctions, the Xiaochaihu, a combination of seven TCMs, is reported to regulate immunity metabolism and oxidative stress [83]. Further, the modified Xiaochaihu decoction could ameliorate age-associated NAFLD by downregulating mRNA/protein levels of core targets in lipid metabolism and inflammation-related pathways such as fatty acid synthase, acetyl-CoA carboxylase, IL-6, and nuclear factor-*β* [84]. Both abovementioned studies are an indication of the therapeutic role of Xiaochaihu decoction against the pathophysiological duo of NAFLD and DM.

In a seminal study, a combined ShengMai-Yin and Ganmaidazao decoction (SGD) showed pharmacological efficacy against T2DM with NAFLD in mice by retarding serum levels of glucose, total cholesterol, triglycerides, free fatty acids, adipocyte size, and liver lipid deposits [85]. Further, SGD can improve liver metabolism through elevating the levels of PPAR*α*, HSL, and PI3K/Akt and decreasing sterol regulatory element-binding protein-1 and fatty acid synthase, resulting in reduced lipid biosynthesis and increased insulin sensitivity. In a rat model of T2DM and NAFLD, another tangganjian decoction efficiently controlled lipid and glucose metabolism by regulating insulin receptor substrate (IRS) and phosphatidylinositol 3-kinase (PI3K)/protein kinase B (Akt) signaling pathways [86].

### 3.7. Berberine

Berberine, a kind of isoquinoline alkaloid obtained from dry roots of *coptidis rhizome*, has been demonstrated to suppress insulin resistance and triglycerides in the liver of NAFLD rats by upregulating levels of IRS-2 [87]. It targets sirtuin 3 (SIRT3)/adenosine 5′-monophosphate- (AMP-) activated protein kinase (AMPK)/acetyl-CoA carboxylase (ACC) and ameliorate progression of NAFLD [88]. It is of note that in diabetic mice, berberine could activate AMPK and regulate lipid metabolism [89]. These two studies indicate the dual therapeutic actions of berberine against NAFLD and DM. In an important report, berberine also mediated its NAFLD-associated therapeutic effect by inhibiting reactive oxygen species (ROS), inflammation, lipid accumulation, tumor necrosis factor-alpha (TNF-*α*) expression, and phosphorylation of nuclear factor kappa B (NF-*κ*B) p65 [90]. Further, berberine can inhibit liver triglyceride synthesis and activate AMP-activated protein kinase (AMPK) and sterol regulatory element-binding protein-1c (SREBP-1c) pathway leading to attenuated hepatic steatosis [91].

### 3.8. Other Herbal Species

Among various TCMs, the *Amomum xanthioides* is an important herb with hepatoprotective, gastrointestinal protection, and antidyslipidemic effects [92]. The administered ethyl acetate extracts of *Ammonium xanthioides* in high-fat induced NAFLD mice suggested that the extract may efficiently regulate the weight of adipose tissue and lipid profiles by targeting lipid metabolic markers such as SREBP-1, PPAR-*α*, and AMP-activated protein kinase [93]. Additionally, *Trapa quadrispinosa*, a TCM with an antidiabetic effect, has been found effective in subduing NAFLD through targeting signaling pathways of insulin resistance and lipid metabolisms such as AMP-activated protein kinase (AMPK)/acetyl-CoA carboxylase (ACC)/sterol regulatory element-binding protein (SREBP)/insulin receptor substrate-1 (IRs-1) and protein kinase B (Akt) [94]. Supporting the above studies, the extract of *Lonicera caerulea* suppressed lipid biosynthesis and triglyceride accumulation in both NAFLD mice and HepG2 hepatocyte cell line by activating AMPK/ACC signaling pathways [95]. The seed coat of *Euryale ferox*, a traditional oriental medicine rich in polyphenol, has been found effective to reduce lipid accumulation, oxidative stress, and liver injury through regulating the expression of malondialdehyde, alanine aminotransferase, aspartate aminotransferase, IRS-1, CYP2E1, and superoxide dismutase in mouse model of high-fat-induced NAFLD [96]. In streptozotocin-induced diabetic rats, the extract of *Euryale ferox Salisb* effectively increased the enzymatic activity of superoxide dismutase (SOD), catalase (CAT), glutathione peroxidase (GPx) and reduced glutathione (GSH), and normalized lipid profile [97]. The presence of 2*β*-hydroxybetulinic acid 3*β*-caprylate and pentacyclic triterpene in *Euryale ferox* Salisb has been found responsible for an antidiabetic, antioxidant, and protective role for hepatocytes and pancreatic cells [98–100]. Likewise, the extract of *Folium Mori* has been demonstrated to efficaciously control hyperglycemia, hyperlipidemia, and insulin resistance by regulating IRS-1/PI3K/Glut-4 signaling pathway in diabetic mice [101]. Collectively, this accumulated body of evidence implies the potential of TCM in offering relief from concomitant liver disease and DM.

## 4. Innovative Avenues in Regenerative Therapy

### 4.1. Stem Cell-Based Repair and Regeneration

**Figure 2:**
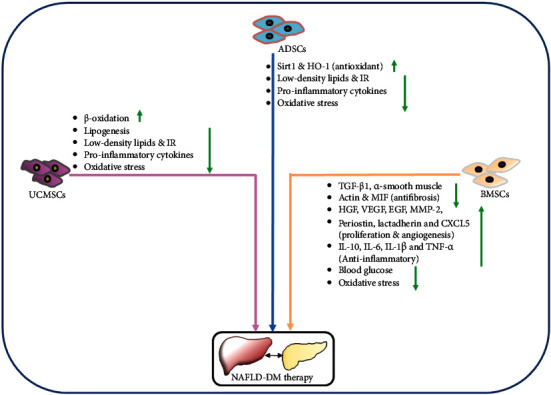
Stem cell-based regenerative therapies for NAFLD in T2DM. (a) BMSC, ADSC, and UCMSC-based regenerative therapies. NAFLD: nonalcoholic fatty liver disease; T2DM: type 2 diabetes mellitus; BMSCs: bone-marrow stem cells; ADSCs: adipose-derived stem cells; UCMSCs: umbilical cord-derived stem cells; HO-1: heme oxygenase-1; IR: insulin resistance; TGF: transforming growth factor; IL: interleukin; HGF: hepatocyte growth factor; VEGF: vascular endothelial growth factor; EGF: epidermal growth factor; MMP-2: matrix metalloproteinase-2.

The application of stem cells in addressing the repair and regeneration of injured tissues is accountable for their differentiation potential into target cells under specific conditions. Of various stem cell types, the bone marrow stem cells (BMSCs), adipose-derived stem cells (ADSCs), and umbilical cord-derived stem cells (UCMSCs) have been employed for diabetes-associated disorders (Figure 2).

**Figure 3:**
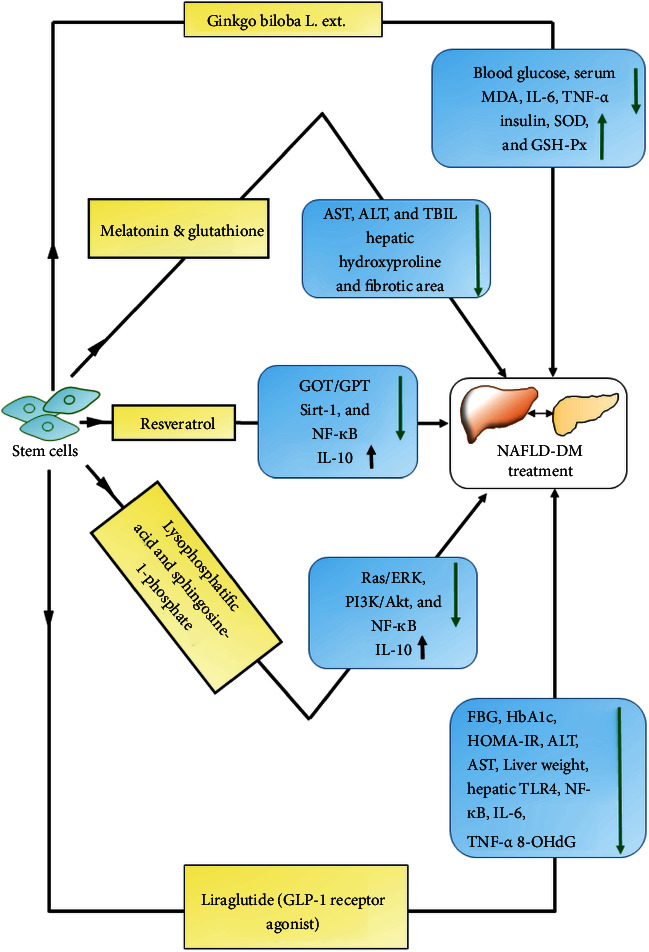
Highlights of novel therapeutic approaches by combining stem cells with the various therapeutic agent (yellow boxes) such as *Ginkgo biloba* extract (TCM), antioxidants (melatonin, reduced glutathione, and resveratrol), bioactive lysophospholipids (lysophosphatidic acid and sphingosine-1-phosphate), and glucagon-like peptide-1 receptor agonist (liraglutide) and their impacts (blue boxes). Up and down arrows indicate the increased and decreased levels, respectively. MDA: malondialdehyde; IL-6: interleukin-6; TNF-*α*: tumor necrosis factor-*α*; SOD: superoxide dismutase; GSH-Px: glutathione peroxidase; ALT: alanine aminotransferase; AST: aspartate aminotransferase; TBIL: total bilirubin; GOT: glutamic-oxaloacetic transaminase; GPT: glutamic-pyruvic transaminase; NF-*κ*B: nuclear factor kappa beta; IL-10: interleukin-10; ERK: extracellular signal-regulated kinases; PI3K: phosphatidylinositol 3-kinase; AKT: protein kinase B; FBG: fasting blood glucose; HbA1c: hemoglobin A1C; HOMA-IR: homeostatic model assessment of insulin resistance; TLR4: Toll-like receptor 4; 8-OHdG: 8-hydroxydeoxyguanosine (oxidative stress marker).

Additionally, stem cells have been synergistically applied with different treatment approaches such as TCMs and oxidative agents (Figure 3).

### 4.2. BMSC-Mediated Therapeutic Bioengineering

In diabetic mice, the BMSC and BMSC-conditioned medium have been shown to repair and regenerate damaged hepatocytes by reducing the infiltration of bone marrow-derived cells, lipid accumulation, insulin resistance, and expression of proinflammatory cytokines [102]. The transplanted MSCs in diabetic mice may suppress fatty liver states by reducing low-density lipids and inflammatory cytokines and elevating Sirt1 and heme oxygenase-1 levels [103]. BMSCs through their paracrine actions may increase the levels of heme oxygenase-1 resulting in a reduction in neutrophil influx, inflammation, and hepatocyte apoptosis [104]. These cells also possess the capacity to reverse weight gain, expansion of subcutaneous adipose tissue, and inhibit steatosis, lobular inflammation, fibrosis via immunomodulation, and immunosuppression, including the suppression of CD4+ T cells [105]. Therefore, BMSCs have been suggested to possess the clinical potential for the treatment of NAFLD. Interestingly, a seminal study showed that supplementation of *Ginkgo biloba L*. extract during BMSC therapy could reduce oxidative stress and blood glucose levels of diabetic rats [106]. This research indicates that a combinatorial approach of cellular therapies and TCMs could offer an improved therapeutic efficacy.

### 4.3. ADSC-Mediated Therapeutic Bioengineering

Compared to BMSCs, the ADSCs are the preferred choice owing to their ease of isolation and comparable efficacy. These have also been explored in addressing regenerative therapeutic needs for liver fibrosis, NAFLD, and liver cirrhosis [107–110]. In the T2DM rat, the transplanted ADSCs assuaged hyperglycemia and insulin resistance as well as liver fibrosis through suppressing TGF-*β*1 levels and phosphorylation of SMAD3 [107]. ADSCs may also ameliorate liver fibrosis by upregulating hepatocyte growth factor (HGF) and downregulating levels of *α*-smooth muscle actin [111, 112]. Apart from monotherapy of ADSCs, their treatment with antioxidants such as melatonin and glutathione strongly inhibits oxidative stress and liver fibrosis [113]. In the rat model of T2DM and liver fibrosis, an elevated reparative and regenerative influence of ADSCs was found with oral consumption of resveratrol, which was confirmed through reduced oxidative damage and enhanced survival signaling [114]. Moreover, the regenerative effect of human ADSCs can be improved by cotreatment of lysophosphatidic acid and sphingosine-1-phosphate in the terms of attenuated histologic damage, suppressed oxidative stress, inflammation, fibrosis, and lipid metabolism dysfunction, without tumor formation [115]. As NAFLD and other diabetic complications are associated with hyperglycemia-induced inflammatory effect, the infusion of ADSCs in diabetic rats has shown anti-inflammatory actions by secreting cytokine IL-10, IL-6, IL-1*β*, and TNF-*α* [116]. ADSCs could promote proliferation and angiogenesis of hepatocytes by secreting growth factors such as HGF, VEGF, EGF, MMP-2, periostin, lactadherin, and CXCL5 [117]. These cells could also decelerate liver fibrosis by secreting macrophage migration inhibitory factor (MIF) and may regenerate the liver by attenuating acute rejection and reducing inflammatory responses [118]. Remarkably, brown adipose tissue also possesses the potential to lower blood glucose/lipid and suppress oxidative stress and fibrosis and improves lipid metabolism in diabetic mice [119]. This could be achieved by downregulating liver metabolic genes and elevating miRNA-99a through negatively regulating the expression of NOX4. On contrary, a clinical study indicated that though transplanted autologous MSCs, T2DM patients were able to improve the liver function and insulin resistance; the diabetic condition remained unaffected [120].

### 4.4. UCMSC-Mediated Therapy

In addition to ADSCs and BMSCs, stem cells derived from UCMSCs have also been explored to develop regenerative therapeutic approaches for diabetes and liver-related disorders. In a mouse model of T2DM and NAFLD, the UCMSCs significantly lowered the lipid and LDL content by regulating lipid metabolism genes leading to promoted *β*-oxidation and suppressed lipogenesis [121]. Moreover, the synergistic application of liraglutide (glucagon-like peptide-1 receptor agonist) and h-UCMSCs may reduce inflammation and oxidative stress through regulating the TLR4/NF-*κ*B inflammation pathway in SD rats with NAFLD and T2DM [122]. These results are also an indication of improved lipid metabolism, insulin resistance, and suppressed liver injury.

It is important to note that regenerative therapeutic efficacy depends on the appropriate homing of injected cells in target tissue or organs. This had been manifested in T2DM mice which showed antidiabetic and antidyslipidemic effects of administered h-UCMSCs with improved liver function migrated after homing to the liver as well as pancreatic islets [123]. Further, the h-UCMSCs-derived exosomes also may improve the structural and functional status of the fibrotic liver through their antifibrotic activity via downregulating the expression of collagen (types I and III) and TGF-*β*1 [124]. Furthermore, the clinical potential of infused h-UCMSC has already been validated through their hepatoprotection and antiviral activity in end-stage liver disease patients without any adverse reactions [125]. A clinical phase I/II study reported that cord blood-derived stem cells could modulate the immune response and restore cytokine balance in T2DM patients [126] and hence could improve insulin resistance mediating concomitant liver disorder and DM. Though the previously discussed stem cells have demonstrated numerous therapeutic outcomes, adverse reactions should be carefully considered before clinical applications. In an important study, a diabetic mouse transplanted with ESCs-derived insulin-secreting cells lowered the glucose level, however, resulted in teratoma formation, which limits its clinical potential in addressing diabetes and related complication [127]. Besides this, mitigating the growth of drug-resistant cancer stem cells in diabetes patients is a major challenge for regenerative therapy [128]. Thus, more preclinical and clinical studies are required to completely establish the role of stem cells in providing a safe and effective alternative for liver disease under diabetic conditions.

### 4.5. Scope of Platelet-Derived Biomaterial (PDB) Therapy in Liver Disease with DM

**Figure 4:**
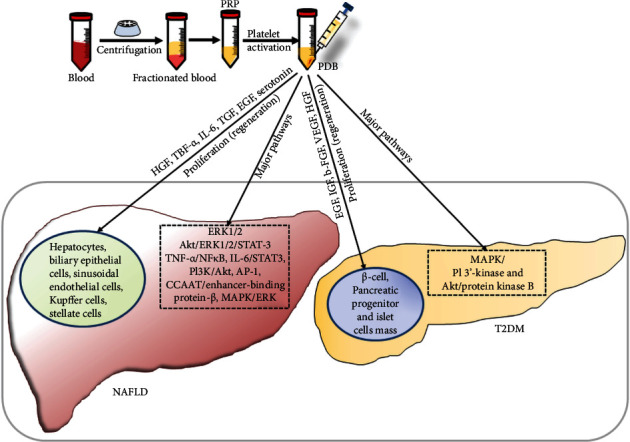
Association between a pathophysiological duo of NAFLD and DM and its treatment. (a) The mechanistic insight underlying NAFLD and DM. NAFLD participates in the development of T2DM by enhancing glucose production and insulin resistance in the liver. T2DM and systemic insulin resistance induce the initiation and progression of NAFLD by increasing levels of FFA and TG from peripheral tissues to the liver. If remain untreated, NAFLD further progresses from NASH, cirrhosis, to hepatocellular carcinoma. (b) Journey of therapeutic alternatives from traditional to regenerative medicines, including lifestyle management, TCM, pharmaceuticals, stem cells, and PRP. NAFLD: nonalcoholic fatty liver disease; FFA: free fatty acid; TG: triglycerides; NASH: nonalcoholic steatohepatitis; T2DM: type 2 diabetes mellitus; TCM: traditional Chinese medicines; PRP: platelet-rich plasma.

Owing to its contained PDBs, the platelets have played a significant role in regenerative medicine. These PDBs are present in platelet's *α*-granules in the form of epidermal growth factor (EGF), platelet-derived angiogenesis factor (PDAF), vascular endothelial growth factor (VEGF), insulin growth factor (IGF), platelet factor-4 (PF-4), transforming growth factor-*β* (TGF-*β*) along with other releasates such as fibronectin, and vitronectin promote cellular regeneration (Figure 4) [129, 130]. PDB may stimulate vascularization, angiogenesis, fibroblast differentiation, and graft adhesion and improve microenvironment and epithelialization leading to wound healing [131]. The platelets hasten liver regeneration by stimulating the proliferation of hepatocytes, biliary epithelial cells, liver sinusoidal endothelial cells, Kupffer cells, and hepatic stellate cells [132]. This is mainly achieved by intercellular interactions between various growth factors and cytokines, such as HGF, tumor necrosis factor-*α*, interleukin-6, TGF, and EGF. The antifibrotic activity of platelets is mediated by deactivating hepatic stellate cells through adenosine-cyclic adenosine 5′-monophosphate signaling pathway. Platelets also inhibit hepatocyte apoptosis by downregulating *Akt* and upregulating *Bcl-xL* signaling pathways, respectively.

As per reports, the majority of PDBs contribute to restoring homeostasis, wound healing, and tissue regeneration via stimulating Akt, extracellular signal-regulated kinase (ERK) 1/2, and IL-6 leading to activation of signal transducers and activator of transcriptions-3 (STAT-3) [133]. The PDBs such as HGF, IGF-1, and VEGF play a crucial role in hepatocyte proliferation through activating Akt/ERK1/2/STAT-3 signaling pathways [134]. Similarly, platelet-derived serotonin may participate in liver regeneration by stimulating the proliferation of hepatocytes or facilitating the release of growth factors IL-6 at the site of liver injury [135]. Moreover, PDBs could stimulate a cascade of transcription factors and associated signaling pathways (TNF*α*/NF-*κ*B, IL-6/STAT3, PI3K/Akt, AP-1, CCAAT/enhancer-binding protein-*β*, and MAPK/ERK) and induce the proliferation of hepatic cells [136]. Platelet-mitigated liver fibrosis occurs through secretion of adenosine which inactivates hepatic stellate cells due to an increase in intracellular cAMP resulting in downregulation of collagen expression [137]. Platelets may also interact with Kupffer cells and trigger the release of IL-6 and TNF-*α* which initiates hepatocyte proliferation [133, 138, 139]. This synergy of Kupffer cells and platelets may effectively increase the efflux of regenerative factors in mouse livers [139, 140]. Platelet-derived-extracellular vesicles such as exosomes may play a crucial role in maintaining cellular homeostasis and liver regeneration by releasing promitogenic factors such as IL-6, which stimulates hepatocyte proliferation [141, 142]. In recent years, PDBs have also gained attention from the scientific and clinical community due to their potential to address diabetes and associated complications. In albino rats, platelet-rich plasma (PRP) has been demonstrated to elevate regeneration of *β*-cell and improved pancreatic islet cell mass [143]. This could be mainly attributed to the release of peptide growth factors such as EGF and IGF which induce mitogen-activated protein kinase- (MAPK-) mediated differentiation of acinar and ductal cells into pancreatic islets [144, 145]. Further, it has been indicated that the encapsulation of *β*-cell into alginate and poly-L-histidine beads supplemented with PRP improves *β*-cell viability and insulin secretion [146]. These outcomes may facilitate the generation of more functional implants with primary *β*-cells or pancreatic islets for DM treatment. The PDBs in the forms of cytokines and signaling molecules could enhance the differentiation potential of stem cells into insulin-secreting cells, which may inhibit insulin resistance. The b-FGF and EGF may promote differentiation of stem cells into islet-like cells and proliferation of Pdx1-positive pancreatic progenitors' cells, respectively, and eventually increase insulin levels [147, 148]. The *β*-cell proliferation, islet number, *β*-cell mass, and total insulin secretion (2-fold) could be increased by overexpression of HGF [149], whereas, VEGF-A and islet vascular structure are correlated and important for the expansion of beta-cell mass [150]. Based on the above reports, we infer that PDB could synergistically act on DM as well as NAFLD by restoring insulin secretion and reducing the risk of initiation and progression of liver-associated disorders by suppressing insulin resistance. Therefore, extensive preclinical and clinical studies should be conducted to establish the dual role of PDB for managing concomitant NAFLD and DM.

## 5. Future Prospects and Conclusion

The pathophysiological association between concomitant liver disorder and DM is highly complicated and therefore poses a challenge in establishing an efficacious therapy. Lifestyle management seems a critical factor to not only reduce glucose production and insulin resistance in the liver but also the systemic insulin resistance caused by T2DM. The TCMs may impart a significant therapeutic impact by suppressing triglyceride synthesis and oxidative stress. Besides, pharmaceutical interventions such as thiazolidinediones or glitazones have been explored, which could benefit both NAFLD and DM by maintaining glucose and lipid homeostasis and suppressing inflammation and liver fibrosis. However, it seems that recent developments in regenerative alternatives including stem cells and platelet-derived biomaterials may provide enhanced therapeutic recovery, owing to their differentiation potential to hepatic and pancreatic lineage. These biological agents could not only suppress hyperglycemia and insulin resistance but also dyslipidemia. Recently explored, the synergistic application of stem cells with TCMs (Ginkgo biloba extract), antioxidants, PDBs, and other bioactive molecules (liraglutide) seems to possess high potential to treat the comorbid state of NAFLD and DM, by combined repairing and regenerative modalities. However, these available pieces of evidence should be extensively investigated for their optimized procedure, efficacious dosage, clinical application, and their safety.

## Data Availability

No data were used to support this study.
